# Incidence of Cerebral Infarction in Northwest China From 2009 to 2018

**DOI:** 10.7759/cureus.17576

**Published:** 2021-08-30

**Authors:** Yu-Xuan Shang, Lin-Feng Yan, Elyse M Cornett, Alan D Kaye, Guang-Bin Cui, Hai-Yan Nan

**Affiliations:** 1 Department of Radiology & Functional and Molecular Imaging Key Lab of Shaanxi Province, Fourth Military Medical University, Shaanxi, CHN; 2 Department of Anaesthesiology, Louisiana State University (LSU) Health Shreveport, Shreveport, USA

**Keywords:** stroke, cerebral infarction, epidemiology, cerebrovascular disease, distribution

## Abstract

Background: There is a lack of epidemiological analysis of patients with cerebral infarction in northwest China. In the present investigation, we conducted a retrospective analysis to collect information on epidemiological characteristics of patients with cerebral infarction in five provinces of northwest China and the Shanxi Province of patients who were hospitalized in the Tangdu Hospital. This project should provide a scientific basis for active prevention and treatment of cerebral infarction.

Material and methods: A retrospective analysis of patients with epidemic characteristics of cerebral infarction that were admitted to the Tangdu Hospital of northwest China from January 2009 to December 2018.

Results: A total of 18,302 patients (aged 1-97 years) with confirmed cerebral infarction, including 12,201 males and 6,101 females, were retrospectively enrolled in this study. The most common lesion site was the cerebellum (51.5%). The incidence of cerebral infarction was slightly higher in workers and laborers, favoring male patients and those aged 40-70 years. The difference between men and women gradually increased after the age of 30.

Conclusions: In this study, 18,302 hospitalized patients with cerebral infarction from different occupations were included. Those engaged in physical labor were more likely to have a cerebral infarction. The incidence of cerebral infarction in males was higher than in females. Cerebellar and cerebral area infarctions were the most common.

## Introduction

Stroke is a severe form of cerebrovascular disease caused by the interruption of blood supply to the brain [1,2]. During a stroke, oxygen and nutrients cannot reach the brain due to the rupture of blood vessels or clots, which causes brain tissue damage. Stroke can occur rapidly and has a high disability rate and mortality rate [3,4]. According to epidemiological surveys, there are 1.5 - 2 million new stroke cases in China every year [5,6]. Of these cases, 70% are cerebral infarctions with corresponding high morbidity and mortality [7,8]. Of note, hypertension is significantly associated with the incidence and mortality of stroke in these populations [9,10]. In other studies [11,12], the elderly suffer from cerebral infarction, while young patients become vulnerable to cerebral infarction [13]. However, older patients are at a greater risk of cerebral infarction than younger patients. With age, arteries naturally become narrower and harder and are more likely to become clogged with fatty material (atherosclerosis). Some research indicates that the occurrence of cerebral infarction increases every five years after the age of 40, and the average onset age of cerebral infarction is 60 years old [14]. Several previous studies suggested that the incidence of cerebral infarction rises with the increase of age [15]. A 2005 study in China showed that the incidence of cerebral infarction was higher in northern cities than in southern cities, and that patients with cerebral infarction in some Chinese cities were older than foreigners in western cities [5]. A 2010 study in Tibet, China, revealed that the age of cerebral infarction decreased. Among the patients with first cerebral infarction, those younger than 45 years old accounted for 12.8%, higher than that in other studies [16]. However, in the past decade, patients' epidemiological characteristics with cerebral infarction in Northwest China are still unclear. Therefore, we retrospectively enrolled cerebral infarction patients in a tertiary hospital with the hope of revealing the epidemiological characteristics of cerebral infarction patients in northwest China, to understand the trend of cerebral infarction incidence during the past decade, and analyze the age or gender difference of cerebral infarction incidence.

## Materials and methods

Ethics statement

The ethical approval of this study was waived because of its retrospective nature.

Date source

This study was a retrospective investigation and in compliance with the national China guidelines. The diagnostic criteria for the clinical diagnosis of cerebral infarction cases are based on the diagnostic criteria for various cerebrovascular diseases in 2013.

Inclusion and exclusion criteria

We collected patient data and excluded: (1) multiple lacunar cerebral infarction and lacunar cerebral infarction; (2) patients with fracture and hematoma; (3) cerebral hemorrhage; (4) malignant tumors.

Data collection and management

According to the new cerebrovascular disease classification, cerebral infarction inpatients in Tangdu hospital were screened and collected. All the data were stratified according to the following four factors: gender, age (<40 vs.≥40 years old), onset time (spring, summer, autumn, and winter), occupation. Categorical variables were summarized as frequency counts and percentages.

Epidemiological characteristics

Incidence was calculated based on the number of confirmed cerebral infarctions and the total number of hospitalizations in the corresponding year (2009-2018). The age distribution of cerebral infarction cases was analyzed in a 10-year-bin group. To describe the geographic distribution of cerebral infarction patients in northwest China, we collected the areas where the patients came.

Statistical analysis

Comparisons between two or more groups of variables were performed using Fisher’s exact test. A P value of <0.05 was considered statistically significant. All statistical analyses were performed using Statistical Package for Social Sciences (SPSS) software, version 20.0 (SPSS Inc. Chicago, IL, USA).

## Results

1. Incidence

**Figure 1:**
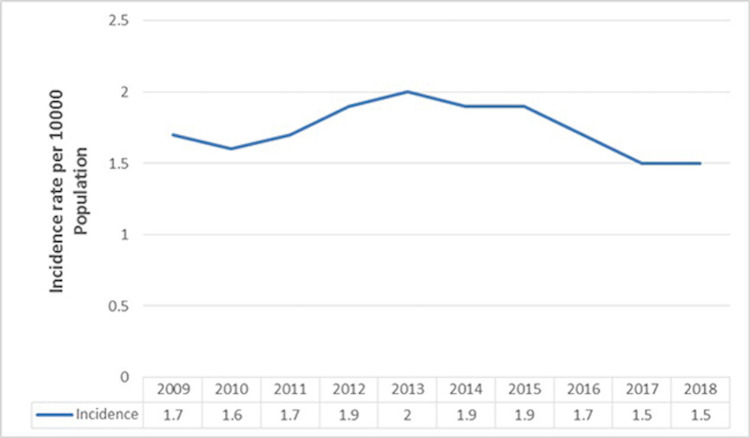
The annual incidence (per 10,000-person year) of cerebral infarction based on hospital patient population during the past decade (2009–2018).

**Figure 2:**
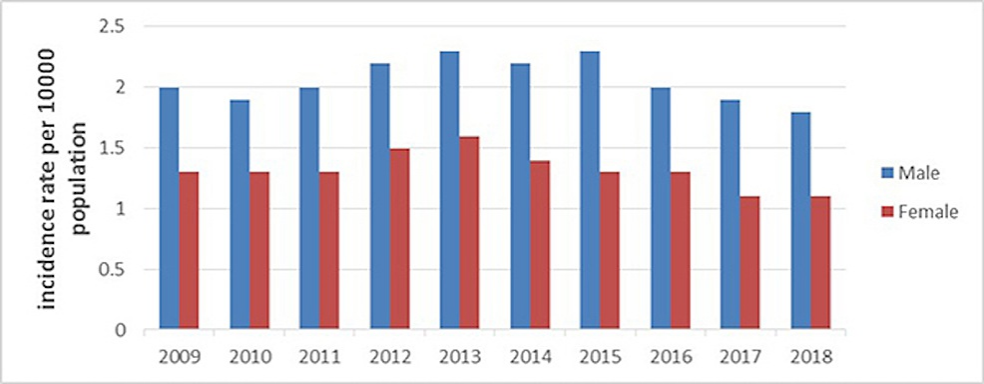
The female and male incidences (per 10,000-person year) of cerebral infarction during the past decade (2009–2018).

A total of 18,302 patients with cerebral infarction from 2009 to 2018 were collected to analyze the annual incidence (including sex-specific incidence) based on the hospitalized patient population. From 2009-2018, the overall incidence of cerebral infarction increased to reach a peak of 2.0 cases per 100,00 patients in 2013 and then decreased until 2017. From 2009 to 2018, the incidence rate in men was higher than that in women. The increased tendency in women was modest, while men's incidence during that period is not stable (Figures 1-2).

2. The location

**Figure 3:**
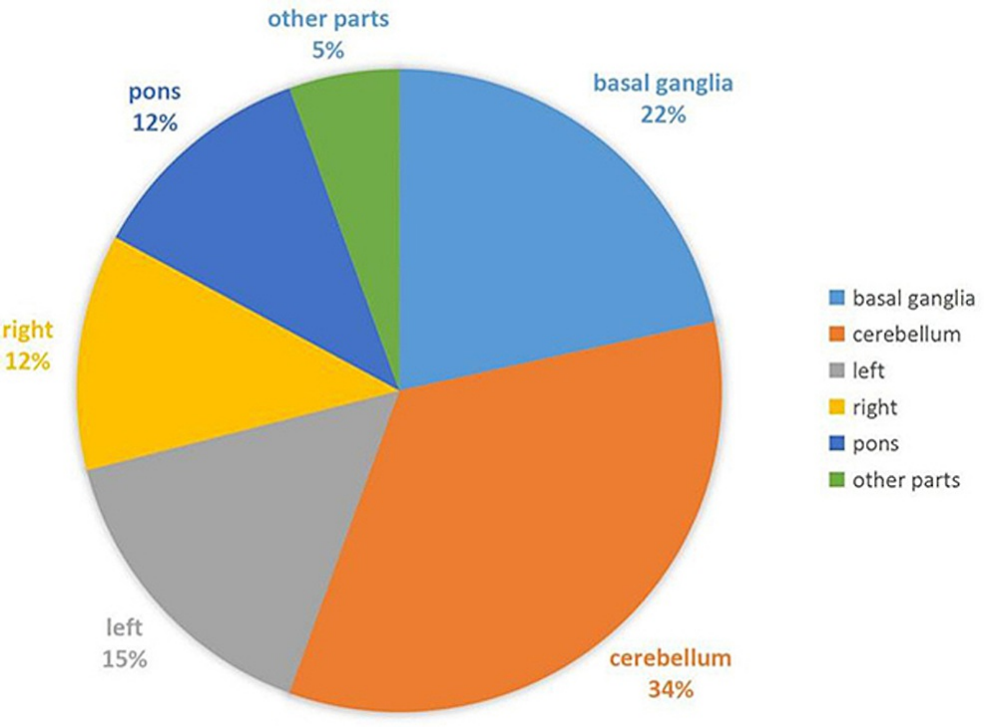
The location of cerebral infarction, 34% of the infarcts were located in the cerebellum and 22% in the basal ganglia.

The common sites of cerebral infarction are basal ganglia, cerebellum, left hemisphere, right hemisphere, and pons. According to our review of patients admitted to Tangdu hospital with cerebral infarction from 2009 to 2018, the cerebellum (34%) is the most common site of patients admitted with cerebral infarction in our hospital, followed by basal ganglia (22%) and left and right hemispheres (Figure 3).

3. Age and season

**Figure 4:**
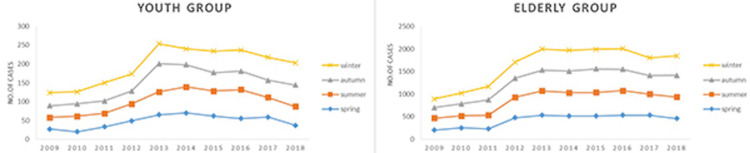
Seasonal distribution of cerebral infarction patients in the youth group and elderly group

Patients were divided into the youth group (below 46 years and a total of 1,924) and the elderly group (age 46 and above, a total of 1,6378). The incidence was highest in both the youth and the elderly groups in winter and lowest in spring. The number of infarctions in the youth group increased significantly from 2011 to 2013, while the number of the elderly group increased from 2012 to 2013, but then stabilized (Figure 4).

4. Occupation

**Figure 5:**
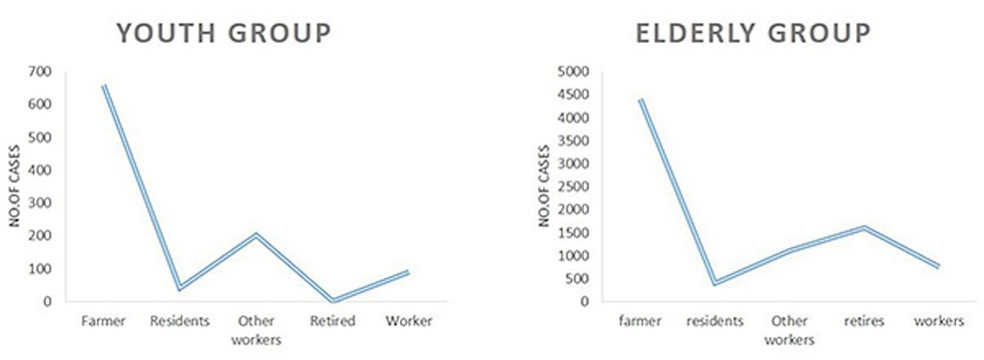
Occupational distribution of cerebral infarction patients in the youth group and the elderly group.

**Figure 6:**
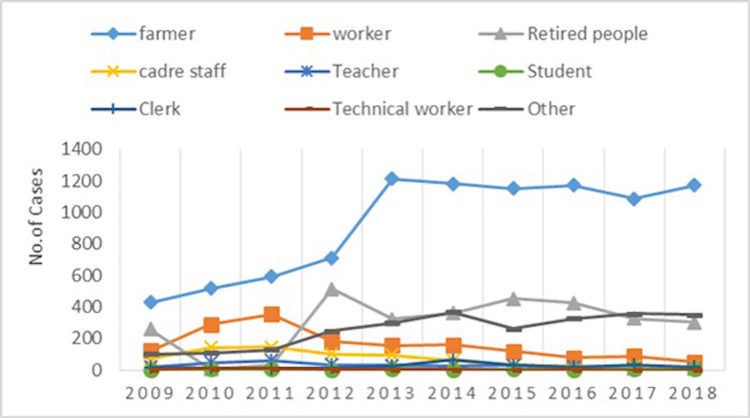
The number of cases in different occupations from 2009-2018.

The relationship between cerebral infarction and occupation was investigated in the young (below 46 years and, a total of 1,924) and old (46 years old and above, a total of 1,6378) groups. The incidence of cerebral infarction in farmers, workers, retirees, small groups, teachers, students, staff, other workers, and technicians were calculated and compared. Farmers, workers, or other occupations engaged in heavy labor had a higher incidence of cerebral infarction than other occupations including, students, teachers, clerks, retired people, or technicians (Figures 5-6).

5. The geographical distribution

**Figure 7:**
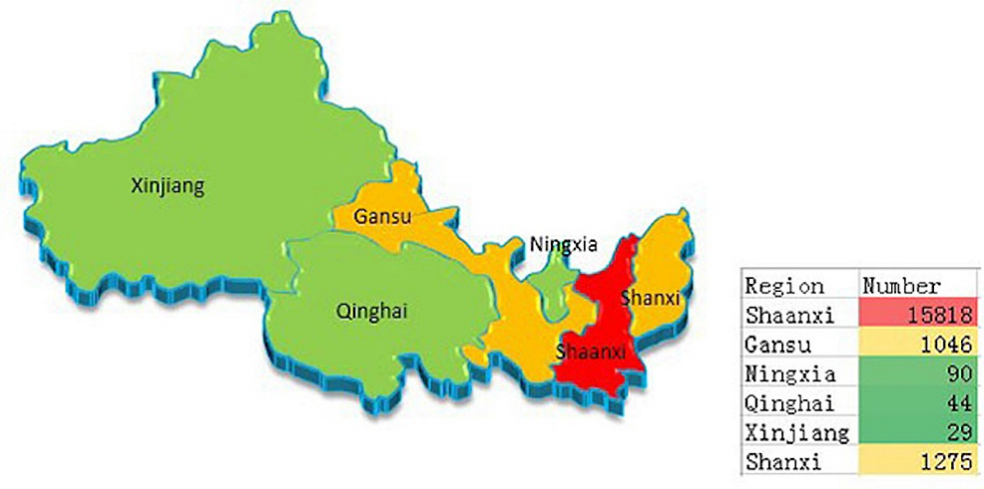
The number of hospitalized patients with cerebral infarction in five provinces in northwest China and Shanxi Province.

The geographical distribution of cerebral infarction patients is presented in Figure 7 and shows the number of hospitalized patients with cerebral infarction in five provinces in northwest China and Shanxi Province. Most patients were farmers and manual laborers. These patients’ access to medical treatment was not considered sufficient, so they were transferred to our hospital for further treatment.

## Discussion

Overall, our results reveal that the incidence of cerebral infarction gradually increased until 2013, however in northwest China, it decreased slightly from 2009 to 2018. The incidence of cerebral infarction began to increase drastically after the age of 40. The incidence of cerebral infarction was higher in males than in females, and the incidence difference between males and females gradually increased after the age of 40.

The cerebral infarction was mainly located in the cerebellum and basal ganglia, followed by the left cerebral hemisphere, the right hemisphere, and pons. According to two studies in 2005, brain infarction locations are not uniform and occur in the left hemisphere more frequently than the right hemisphere [17-18]. However, in this study, we found cerebral infarction was mainly located in the cerebellum. Therefore, cerebellum infarction may be more common than previously thought. Reporting of cerebellar infarction is likely small because most of these infarctions may be asymptomatic or mild, nonspecific or focal symptoms, such as dizziness, nausea and vomiting, gait instability, and headache [19,20]. Therefore, it is possible that most cerebellar infarcts can evade clinical attention in the acute phase [21].

Studies in counties and climatic regions worldwide have shown an increase in the incidence of cerebral infarction and the number of hospitalizations for cerebral infarction in the cold seasons (winter and spring), compared with the warm seasons (summer and autumn). In Japan, cerebral infarction incidence was the highest in winter and spring [22]. According to an Australian study, cerebral infarction incidence increases from summer to winter [23]. Our survey found that rates of infarction were highest in young and elderly inpatients in the winter and lowest in the spring. The results echo the Australian study and are similar to the study in Japan. However, differences in data may be related to different populations and locations.

A survey in our hospital found that cerebral infarction is more likely to occur in jobs that require more manual labor. For example, farmers and workers are the two types of occupations that are the most prone to cerebral infarction. Most of these two occupational groups come from rural areas, belong to low-income groups, work long hours, lack exercise, have an irregular diet and lifestyle, and are prone to high blood pressure and other diseases, leading to cerebral infarction. According to two studies in 2014 and 2016, the risk of cerebral infarction between urban and rural residents varies greatly [24,25]. Young urban people are more engaged in work involving mental labor and higher income, while rural people are involved in lower-income work and likely have low awareness of disease prevention. Therefore, the risk factors for cerebral infarction in rural areas are much higher than that of urban high-income people. The incidence rate is relatively stable for other occupational groups, and it is more common among the elderly.

This study has some limitations. First, because this study is retrospective, we were unable to retrieve all the necessary information. Second, based on the hospital population, the investigation of the incidence of cerebral infarction in a single center may lead to biased conclusions. To consolidate this conclusion, a multicenter investigation based on general assumptions is needed.

## Conclusions

This retrospective investigation found that cerebral infarction incidence is higher in males than females. People engaged in manual labor are more likely to have cerebral infarction, and the morbidity and mortality of cerebral infarction increase with age. The most common sites of infarction are the cerebellum and basal ganglia. Finally, patients born in the five provinces of northwest China tend to be stable but show a slightly higher incidence of infarction in the summer and the highest incidence of infarction in the winter.
